# Brain-injury and Alzheimer’s disease biomarkers are elevated in patients with suspected infection and physiological derangement: importance for context-specific interpretation of Alzheimer’s biomarkers

**DOI:** 10.1093/braincomms/fcag063

**Published:** 2026-02-28

**Authors:** Daniel P Whitehouse, Jack Cafferkey, Andrew Ferguson, Soraya Ebrahimi, Timothy Rittman, Michael Hornberger, Alasdair Gray, Alasdair R Corfield, Virginia F J Newcombe, Edward J Needham

**Affiliations:** Department of Medicine: Perioperative, Acute, Critical Care and Emergency Medicine (PACE) Section, University of Cambridge, UK; Department of Emergency Medicine, Royal Infirmary of Edinburgh, Emergency Medicine Research Group Edinburgh (EMERGE), Edinburgh, UK; Department of Emergency Medicine, Royal Infirmary of Edinburgh, Emergency Medicine Research Group Edinburgh (EMERGE), Edinburgh, UK; Department of Clinical Neurosciences, University of Cambridge, UK; Department of Clinical Neurosciences, University of Cambridge, UK; Norwich Medical School, University of East Anglia, Norwich, UK; Department of Emergency Medicine, Royal Infirmary of Edinburgh, Emergency Medicine Research Group Edinburgh (EMERGE), Edinburgh, UK; Acute Care Edinburgh, Centre for Population and Health Sciences, Usher Institute, University of Edinburgh, Edinburgh, UK; Emergency Department, Royal Alexandra Hospital, Paisley, UK; Department of Medicine: Perioperative, Acute, Critical Care and Emergency Medicine (PACE) Section, University of Cambridge, UK; Department of Clinical Neurosciences, University of Cambridge, UK; NIHR BioResource, Cambridge University Hospitals, Cambridge Biomedical Campus, Cambridge CB2 0QQ, UK

**Keywords:** biomarkers, cytokines, infection, brain injuries, sepsis

## Abstract

Following acute infective illness, patients frequently exhibit neurological symptoms, with persistent neurological symptoms commonly observed following severe infection. However, the association between systemic infection and the concentration of blood-based biomarkers of brain injury and the relationship between these and markers of the host response to infection are poorly characterized in the literature. Further, the association between acute illness and the Alzheimer’s disease–associated biomarker phosphorylated-tau-217 (p-tau-217) is unknown. In acute samples from 26 patients attending the emergency department with suspected sepsis (clinically suspected or proven infection, a National Early Warning Score or National Early Warning Score 2 score ≥ 5), the levels of serum biomarkers of brain injury (neurofilament light [NfL], glial fibrillary acidic protein (GFAP), total tau and ubiquitin C-terminal hydrolase L1) and p-tau-217 were compared to age- and sex-matched non-infected controls, with further assessment of the correlation between the biomarker levels and cytokine profiles. p-tau-217 levels were additionally compared to a positive control group of patients with diagnosed Alzheimer’s disease. Both total tau (*P* = 0.006, Wilcoxon rank-sum test) and NfL (*P* = 0.044, Wilcoxon rank-sum test) levels were significantly higher in patients with suspected sepsis as compared to controls, with no significant differences in levels of GFAP or ubiquitin C-terminal hydrolase L1. Within suspected sepsis patients, serum total tau levels were associated with multiple cytokines and a summary cytokine score (Spearman’s rank correlation coefficient ρ = 0.65, *P* < 0.001). There were significantly higher p-tau-217 levels in suspected sepsis patients as compared to non-infected controls (*P* < 0.001, Dunn–Kruskal–Wallis test), with no significant difference compared to Alzheimer’s disease controls (*P* = 0.118, Dunn–Kruskal–Wallis test). Of patients with suspected sepsis, 29% had a p-tau-217 level classified as high (>0.63 pg/ml) with a further 17% classified as intermediate (0.4–0.63 pg/ml). In conclusion, we have identified elevated levels of total tau and NfL compared to age- and sex-matched controls, along with significant correlations between these tau levels and cytokine levels. Additionally, we observed elevated levels of p-tau-217 in the patient cohort, with levels comparable to those seen in Alzheimer’s disease patients. Further analysis is required to replicate the findings of this study in larger cohorts. However, the results suggest a potentially extracranial source of tau expression in the context of infection or physiological stress. Given the potential for acute illness to influence p-tau-217 levels, our results raise important considerations regarding the interpretation of p-tau-217 as a diagnostic marker for Alzheimer’s disease in patients with active infection.

## Teaser

Whitehouse et al. report elevations in brain-injury and Alzheimer’s disease biomarkers in patients with suspected sepsis. This exploratory secondary analysis of the ABC-Sepsis study indicates that acute infection and/or physiological stress may elevate biomarker levels, including total tau and p-tau-217, potentially confounding their interpretation in diagnostic or research contexts.

## Graphical Abstract

**Graphical Abstract:**
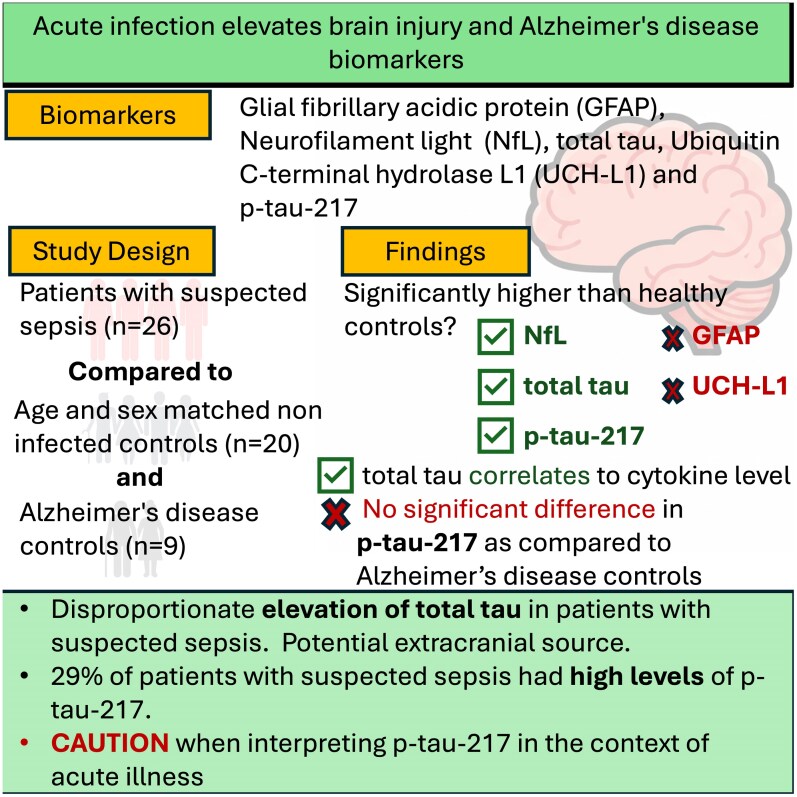

## Introduction

Sepsis is defined as ‘life-threatening organ dysfunction caused by a dysregulated host response to infection’.^1^ Patients who experience severe infection, including sepsis, frequently experience disturbance of brain function (i.e. encephalopathy/delirium).^2^ As the infection resolves, many suffer from persistent neuropsychological deficits, including cognitive impairments, concentration issues and fatigue.^3^

Biomarkers, including the astrocyte intermediate filament protein glial fibrillary acidic protein (GFAP), axonal markers neurofilament light (NfL) and total tau, and the marker of the ubiquitin–proteasome system ubiquitin C-terminal hydrolase L1 (UCH-L1) have all been studied as markers of brain injury.^4^ Although most commonly studied in primary neurological disorders such as traumatic brain injury, these biomarkers have been shown to be elevated in systemic illnesses including COVID-19 infection,^5^ sepsis^6,7^ and sepsis associated encephalopathy,^8^ and to associate with pro-inflammatory cytokines in systemic illness.^5^ While there is some extracranial expression of these biomarkers (for instance, NfL in the peripheral nervous system,^9^ GFAP in the gut^10^ and total tau in the lungs^11^), expression is highly enriched in the central nervous system. As such, elevations in these biomarkers following sepsis are likely to indicate structural brain injury, either from complications of infection or from deleterious neuroinflammation driven by cross-talk from the systemic inflammatory response.^12^

Although there have been prior examinations of these biomarkers in the context of sepsis,^6,7,13^ these have focused on the critical care population, with a lack of comparison to non-infected controls. Most patients attending the emergency department (ED) with suspected sepsis are not admitted to critical care, and evidence of structural brain injury in this population may be of particular interest owing to the volume of patients, and ongoing care outside of an intensive care unit setting. Additionally, no studies have examined the relationship between the acute cytokine levels and brain injury biomarkers in sepsis patients to explore mechanistically whether brain injury relates to the degree of systemic inflammatory response.

Therefore, the primary aim of this study is to assess brain injury biomarkers in patients with suspected sepsis as compared to non-infected controls. We hypothesize that an element of neuronal injury occurs in patients with severe infection, even in the absence of overt neurological signs, and that this will be reflected in elevated brain injury biomarkers. This analysis aims to provide further insight into the extent of brain injury in suspected sepsis and its association with systemic inflammatory responses.

Beyond the acute phase of illness, a dysregulated inflammatory response to infection is thought to be a key risk factor for various neurodegenerative diseases.^14^ It has been hypothesized that infection triggers increased production of protein aggregates, such as amyloid β (Aβ) plaques and neurofibrillary tangles (where tau protein is a major component), that are pathological characteristics of Alzheimer’s disease. When production surpasses clearance, these aggregates accumulate, associating with neuronal loss and cognitive decline.^15^

Certain biomarkers, including tau phosphorylated at different epitopes such as threonine 217 (p-tau-217) or threonine 181 (p-tau-181), have shown accuracy in detecting abnormal Aβ and tau pathologies and are gaining significant traction as diagnostic markers for Alzheimer’s disease.^16,17^ Despite increasing evidence supporting the use of these biomarkers for dementia diagnosis, longitudinal monitoring of disease progression and assessment of treatment response, there is limited knowledge regarding the acute expression profiles of these biomarkers in the setting of common non-neurological diseases, such as infection, compromising the extrapolation of this data into real-life clinical settings.

Acute and chronic p-tau-181 elevations have been described following SARS-CoV-2 infections,^18,19^ but there are no published reports regarding p-tau-217, now the leading candidate biomarker for Alzheimer’s disease diagnosis, in acute infective illness.^20^ Therefore, the secondary aim of this study is to investigate *p*-tau-217 levels in patients with suspected sepsis. We hypothesize that, like *p*-tau-181, *p*-tau-217 may be elevated in the context of acute illness, with implications for the understanding of infection's role in neurodegenerative diseases, but, more pressingly, on the interpretation of these biomarkers in real-world clinical settings.

Overall, this study aims to assess plasma levels of brain injury biomarkers (GFAP, NfL, total tau and UCH-L1) and *p*-tau-217 in patients presenting to the ED with suspected sepsis and to explore their correlations with acute cytokines as markers of the host immune response.

## Materials and methods

### Study populations

Patients studied were from the Mechanistic Inflammatory Sub-study (MIS) (NCT04963569), embedded in the Albumin versus Balanced Crystalloid in Sepsis trial (ABC Sepsis).^21^ MIS-ABC recruited from participants enrolled in ABC Sepsis, a multicentre, open-label, randomized controlled feasibility trial designed to establish the comparative effectiveness of 5% human albumin solution (HAS) as compared to balanced crystalloid for early resuscitation in suspected community acquired sepsis.^21^ The inclusion criteria for ABC Sepsis were adults with clinically suspected or proven infection, a National Early Warning Score (NEWS) or NEWS2 ≥ 5, hospital presentation within the last 12 h, a clinician assessment that intravenous fluid resuscitation is needed and ability to obtain informed consent. The MIS-ABC Sepsis study was an observational study recruiting from the main trial, taking blood samples for immunophenotyping and cytokine analysis and aiming to investigate mechanisms underlying the results from ABC Sepsis. MIS-ABC recruited across three sites: Edinburgh, Paisley and Taunton, with a total of 26 participants included in MIS-ABC between 13 October 2021 and 25 May 2022. Written consent was gained from either patient themselves or from a personal representative if the patient was deemed not to have capacity to consent. Ethical approval for MIS-ABC Sepsis was granted in both England and Scotland separately (21/SS/0052 and 21/NW/0020, respectively).

There were two sources of control samples used in the analysis: (i) age- and sex-matched healthy control plasma samples were sourced from the NIHR BioResource (17/EE/0025) (n = 20) and (ii) plasma of subjects with a diagnosis of Alzheimer’s disease sourced locally from samples collected in a longitudinal transdiagnostic study (The demenTia Research And Care Clinic [TRACC], 16/LO/1366), with numbers (n = 9) selected based on sample availability.

### Clinical information

Demographic, biochemical and clinical information was recorded in the trial case report form from the patient clinical notation recorded by the clinical team. Patient outcome (death or critical care admission) was assessed at 90 days from enrolment using the clinical notation. The age at time of sampling of non-infected controls was supplied by the NIHR BioResource in 10-year age bands.

### Procedures

#### MIS-ABC patients with suspected sepsis

Plasma samples from 26 patients with suspected sepsis were collected at enrolment into MIS-ABC Sepsis. As a secondary analysis, no power calculation was performed with sample size dependent on the availability of samples from the MIS-ABC Sepsis study. Blood samples were taken in the MIS-ABC Sepsis study at three timepoints: initially upon recruitment and then at 12 and 24 h. Only the first sample taken following recruitment was considered in this analysis. Samples were centrifuged at 1100–1300 g for 10 min at room temperature, aliquoted, labelled using pseudonymous study identifiers and stored locally at −80°C until recruitment ended. Samples were then shipped on dry ice to the central biorepository at Edinburgh, Scotland for storage. Cytokine analysis was performed locally at the University of Edinburgh, with samples shipped to Cambridge on dry ice for proteomic biomarker analysis conducted at the University of Cambridge.

### Non-infected controls

Plasma samples from 20 volunteer participants in the NIHR BioResource were selected to provide age- and sex-matched controls. Samples were collected in non-acute research setting at recruitment hubs around the country and transported using 24-h postage at room temperature to the central laboratory. Once samples arrived at the BioResource laboratory, they were centrifuged at 2500 rpm for 10 min at 4°C with the brake and acceleration at nine. The top plasma fraction was removed and aliquoted into FluidX tubes before storage at −80°C in the NIHR BioResource biorepository. Prior to shipment, samples may have undergone one thaw to aliquot the sample to a smaller volume, before being frozen again to −80°C for shipment on dry ice for analysis at the University of Cambridge, UK.

### Alzheimer’s disease controls

Plasma samples from nine subjects with a diagnosis of Alzheimer’s disease were collected in a non-acute research setting, centrifuged at 1300 xg relative centrifugal force for 10 min with samples aliquoted, labelled and stored at −80°C locally in the University of Cambridge, UK.

### Biomarker measurement

GFAP, neurofilament light (NfL), total tau and UCH-L1 were quantified in plasma from non-infected controls and patients with suspected sepsis at the University of Cambridge using the Simoa® Neurology 4-PLEX B assay run on the Quanterix HD-X Analyser. Of the samples analysed using the Neurology 4-PLEX, 10 were excluded owing to assay failure, leaving 36 samples (23 suspected sepsis samples and 13 controls). The analytical lower limit of quantification (LLoQ) for the biomarkers were as follows: GFAP 9.38 pg/ml, NfL 0.5 pg/ml, UCH-L1 9.38 pg/ml and total tau 0.125 pg/ml. No samples were under the LLoQ for GFAP, NFL or total tau. 13% (n = 5) of the UCH-L1 samples were below the LLoQ.

In the suspected sepsis samples, non-infected age- and sex-matched controls and additional ‘positive’ controls taken from Alzheimer’s patients (n = 9), p-tau-217 was quantified using the Simoa® ALZpath p-tau-217 Advantage PLUS Assay Performance run on the Quanterix HD-X Analyser. The analytical LLoQ was 0.00326 pg/mL with no p-tau-217 levels below the LLoQ. Two samples were removed owing to assay failure leaving results of 53 samples (24 suspected sepsis samples, 20 non-infected controls and 9 Alzheimer’s disease controls).

### Cytokine measurement

Concentrations of IL-6, TNF-alpha, GM-CSF, CXCL10, IL-5, IFN-a, IL-4, CCL3, IL-10, IL-12p70, CCL20, IFN-gamma, CXCL8 and CCL4 from the first blood sample taken on recruitment to the study were measured on the Attune NxT flow cytometer using a LegendPlex custom panel at the University of Edinburgh. Cytokine concentrations were derived using the LEGENDplex Qognit platform and included values below the functional LLoQ.

### Statistical analysis

Continuous descriptive data are presented using means and standard deviations (SD), with categorical variables presented using numbers and percentages. Biomarker levels are reported using medians and interquartile ranges (IQR) owing to a non-normal distribution.

Correlations between brain injury biomarker levels in patients with suspected sepsis were assessed using Spearman’s rank correlation coefficient. Biomarker levels in suspected sepsis patients were compared to non-infected controls using the Wilcoxon rank-sum test. Additionally, correlations between biomarker levels and illness severity (assessed by admission lactate and NEWS) were analysed using Spearman’s rank correlation coefficient.

Correlations between plasma cytokine and biomarker levels were analysed using Spearman’s rank correlation coefficient. Adjustment for multiple comparisons per experiment was made using the false discovery rate (FDR) set at 1%, and results were visualized using a volcano plot. A summary score of the cytokine load was created by performing principal component analysis on the scaled cytokine values of participants (n = 26). The score for the first unrotated principal component (PC1) was then used as a summary inflammatory cytokine load score with the biomarker values were then compared to this score using Spearman’s rank correlation coefficient.

p-tau-217 levels were assessed in patients with suspected sepsis, non-infected controls and a ‘positive’ control group of Alzheimer’s disease patients. Correlation between p-tau-217 and total tau levels in suspected sepsis patients was assessed using Spearman’s rank correlation coefficient. Levels of p-tau-217 were compared among three groups (suspected sepsis, non-infected controls and Alzheimer’s disease) using the Kruskal–Wallis test followed by Dunn’s test for groupwise comparisons. p-tau-217 levels were further categorized into low (95% sensitivity, <0.4 pg/ml), intermediate (0.4–0.63 pg/ml) and high (95% specificity, >0.63 pg/ml) in reference to literature-based reference points to rule out or rule in Alzheimer’s disease.^16^ Correlation between p-tau-217 and cytokine levels was analysed using the same methodology as described above. In those with both p-tau-217 and total tau levels (non-infected controls and suspected sepsis patients), the p-tau-217:tau ratio was calculated and compared between the groups using the Wilcoxon rank-sum test. The association between age and p-tau-217 levels within patients with suspected sepsis was assessed using Spearman’s rank correlation coefficient. Demographics, illness characteristics and outcomes between patients with suspected sepsis were compared across p-tau-217 categories using ANOVA tests for continuous variables and a chi-squared test for categorical variables.

Multiple sensitivity analyses were conducted to interrogate the primary findings. Firstly, to assess the impact of ABC Sepsis trial allocation (HAS versus crystalloid), an a priori sensitivity analysis was conducted by comparing biomarker levels between the allocation groups using the Wilcoxon rank-sum test. Secondly, to assess if the observed total tau differences were secondary to pulmonary expression given prior literature demonstrating tau release from pulmonary epithelium,^22,23^ we performed a post-hoc analysis comparing the total tau levels between those with a discharge diagnosis of pulmonary disease (LRTI or pneumonia, community acquired pneumonia, exacerbation of asthma, exacerbation of inflammatory lung disease) and those without using the Wilcoxon rank-sum test. Additionally, we assessed the correlation between fraction of inspired oxygen (FiO_2_) at recruitment and total tau levels using Spearman’s rank correlation coefficient.

All tests were two-tailed, with a *P*-value < 0.05 considered significant unless specified. Data analysis was performed using R version 4.3.2.

## Results

### Demographics

Of the patients with suspected sepsis, 14 were randomized in the ABC Sepsis study to receive HAS, and 12 received balanced crystalloid for early resuscitation. Demographic, clinical and outcome information for the suspected sepsis patients is displayed in Table 1.

**Table 1:** Demographics of the included cohort

		Patients with suspected sepsis (n = 26)	Non-infected controls (n = 20)	Alzheimer’s disease controls (n = 9)
Age (y)		71 (12)	N/A	72
Age (10-year age bands)	<60	4	0	1
	60–70	5	5	2
	70–80	11	10	6
	80–90	4	5	N/A
	90<	2	0	N/A
Sex	Male	12 (46%)	10 (50%)	N/A
	Female	14 (54%)	10 (50%)	N/A
Co-morbidity	COPD	8 (31%)	N/A	N/A
	Diabetes	8 (31%)	N/A	N/A
	Previous myocardial infarction	6 (23%)	N/A	N/A
	Heart failure	5 (19%)	N/A	N/A
	Cancer	5 (19%)	N/A	N/A
	Previous stroke	3 (12%)	N/A	N/A
	Peripheral vascular disease	1 (3.8%)	N/A	N/A
	Chronic kidney disease	1 (3.8%)	N/A	N/A
	Dementia	0 (0%)	N/A	N/A
Lactate at presentation (mmol/l)		2.4 (1.6)	N/A	N/A
Temperature at presentation (°C)		37.6 (1.7)	N/A	N/A
Initial NEWS score (median, range)		7 (5, 12)	N/A	N/A
New confusion at presentation		6 (23%)	N/A	N/A
qSOFA score at presentation	**0**	3	N/A	N/A
	**1**	18	N/A	N/A
	**2**	3	N/A	N/A
	**3**	2	N/A	N/A
Mean length of stay at ward level (days)		13.4 (17.8)	N/A	N/A
Ever admitted to critical care		2 (7.7%)	N/A	N/A
Mean length of stay in critical care (days)		0.5 (1.8)	N/A	N/A
90-day mortality		1 (3.8%)	N/A	N/A
Arrival GCS	15	22 (84.6)	N/A	N/A
	14	3 (11.5)	N/A	N/A
	13	1 (3.8)	N/A	N/A
Confirmed organism on blood cultures	None	21 (80.8)	N/A	N/A
	Escherichia	1 (3.8)	N/A	N/A
	Staphylococcus	3 (11.5)	N/A	N/A
	Streptococcus	1 (3.8)	N/A	N/A
GFAP (pg/ml [Median IQR])		126.00 [77.90, 197.50]	200.00 [102.0, 295.0]	N/A
NFL (pg/ml [Median IQR])		20.10 [13.65, 36.75]	12.10 [11.80, 16.60]	N/A
Total tau (pg/ml [Median IQR])		4.87 [3.16, 8.38]	2.52 [1.85, 3.40]	N/A
UCH-L1 (pg/ml [Median IQR])		30.20 [11.50, 50.80]	43.30 [31.50, 53.00]	N/A

Means (SD) of continuous variables unless otherwise stated, Count (%) of categorical variables. N/A = Not available. COPD = Chronic Obstructive Pulmonary Disease, NEWS = National Early Warning Score, qSOFA = quick Sequential Organ Failure Assessment Score. GFAP, NFL, total Tau and UCH-L1 results available for 23 patients with suspected and 13 controls.

Patients with suspected sepsis had a mean age of 71.3 (SD ± 12.4) with 54% (n = 14) female. The age and sexes of non-infected controls were well matched with the suspected sepsis patients (Table 1). No patients with suspected sepsis had a past medical history of dementia; however, three had a prior history of stroke. Four patients with suspected sepsis presented with new-onset confusion to the ED (three with a GCS of 14 and one with a GCS of 13). No patients had a suspected central nervous system infection. The mean hospital length of stay was 13 days, with one patient dying within 90 days of enrolment, and two patients were admitted to critical care. Five patients had positive blood cultures. Of the suspected sepsis patients with available discharge information (24/26), 22 (92%) had a discharge diagnosis of an infectious illness, with the remainder having a diagnosis of exacerbation of inflammatory lung disease or pulmonary oedema respectively.

### Associations between astrocyte, neuronal and axonal markers

**Figure 1:**
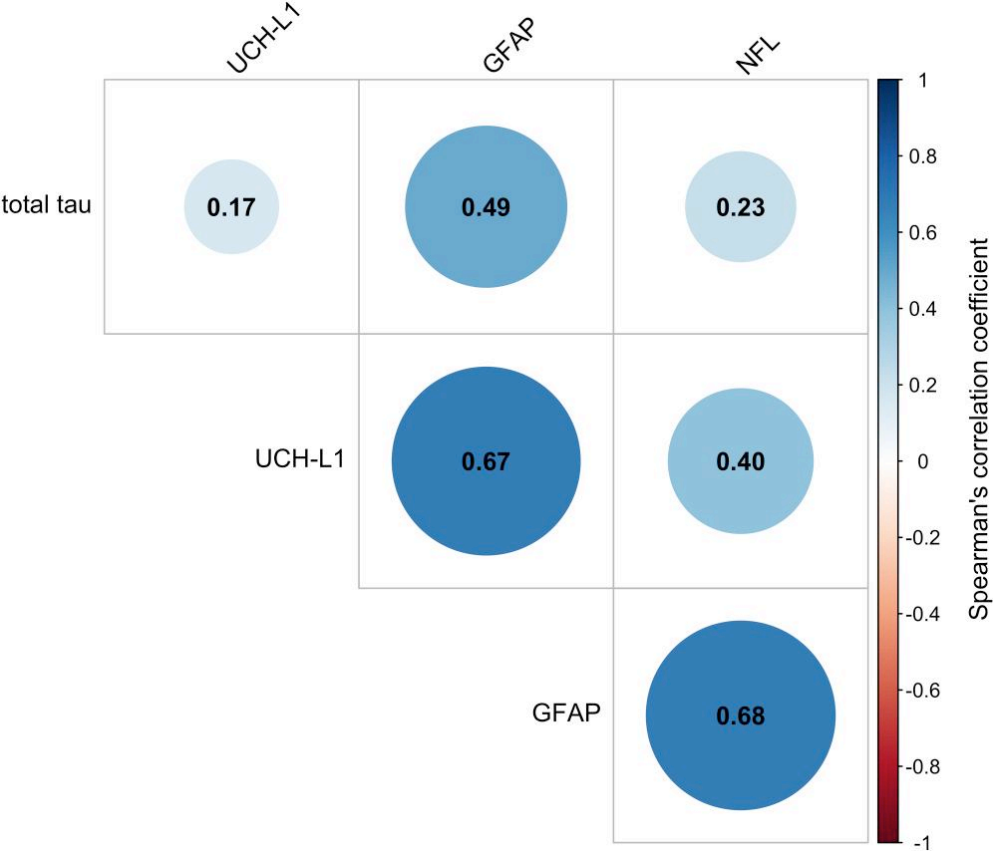
**Correlation plot of the Spearman’s rank correlation coefficient between biomarker levels in patients with suspected sepsis (n = 23).** Correlation matrix of biomarkers in participants with suspected sepsis and complete biomarker levels (n = 23) visualised as a correlogram. Each circle represents the pairwise correlation between two respective biomarkers, with size indicated by the magnitude of the circle, and the colour indicating direction of the correlation (blue = positive, red = negative). The number represents the Spearman’s correlation coefficient. Significant correlations were observed between GFAP and total tau (ρ = 0.49, *P* = 0.017), GFAP and UCH-L1 (ρ = 0.67, *P* < 0.001), and GFAP and NfL (ρ = 0.68, *P* < 0.001). No significant correlations were observed between NfL and UCH-L1 (ρ = 0.40, *P* = 0.062), total tau and UCH-L1 (ρ = 0.17, *P* = 0.450), or total tau and NfL (ρ = 0.23, *P* = 0.293). S100B = S100 calcium-binding protein B.

**Figure 2:**
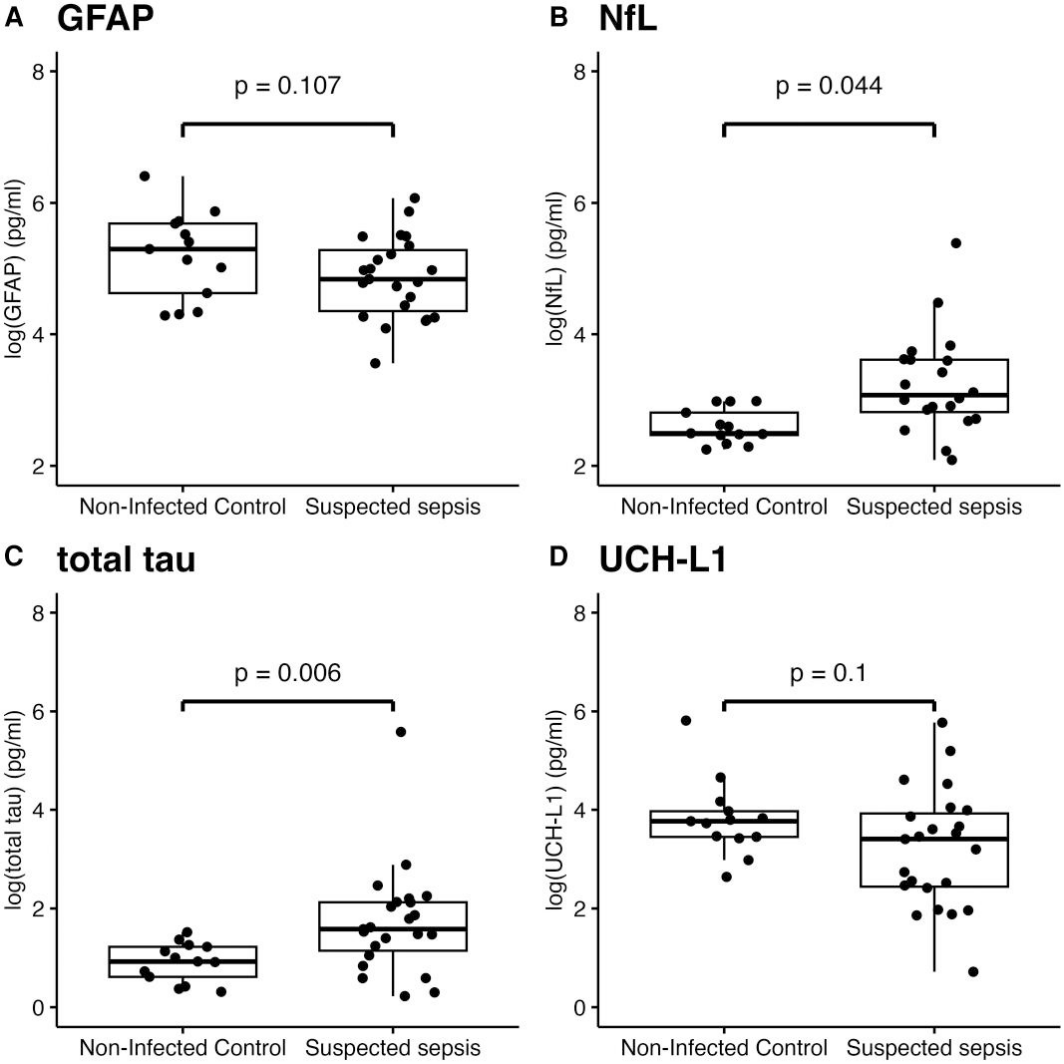
**Boxplots of the biomarker levels in patients with suspected sepsis (n = 23) and age- and sex- matched non-infected controls (n = 13).** Boxplots showing the univariate distribution of biomarker levels in patients with suspected sepsis (n = 23) and age- and sex-matched non-infected controls (n = 13). Panels show: (**A**) log GFAP (pg/ml), (**B**) log NfL (pg/ml), (**C**) log total tau (pg/ml) and (**D**) log UCH-L1 (pg/ml) with each panel corresponding to an individual biomarker. Each box represents the IQR with the median indicated by a horizontal line. Individual participant data points, each representing a single participant’s biomarker measurement, are overlaid as black dots. Horizontal bars indicate statistical comparisons between groups (suspected sepsis versus non-infected controls), with significance (*P*-value) annotated above the bars. Group comparisons were performed using the Wilcoxon rank-sum test: GFAP, W = 199, *P* = 0.107; NfL, W = 88, *P* = 0.044; total tau, W = 65, *P* = 0.006; UCH-L1, W = 200, *P* = 0.100. S100B = S100 calcium-binding protein B.

There was a significant correlation between levels of the astrocyte marker GFAP and: the neuronal marker NfL (ρ = 0.68, *P* < 0.001), the axonal marker tau (ρ = 0.49, *P* = 0.017) and the ubiquitin-proteasome marker UCH-L1 (ρ = 0.67, *P* < 0.001) (Fig. 1). Levels of total tau (W = 65, *P* = 0.006) and NfL (W = 88, *P* = 0.044) were significantly higher in patients with suspected sepsis as compared to age- and sex-matched controls, with no significant difference observed in the levels of GFAP (W = 199, *P* = 0.107) or UCH-L1 (W = 200, *P* = 0.100) between patient groups (Fig. 2).

The median NEWS2 score on recruitment was 7 (range 5–12). 21 patients had a quick Sequential Organ Failure Assessment Score (qSOFA) score of 0/1, with five having a score of 2/3. There was no significant correlation between biomarker levels and either a patient’s NEWS score or lactate on recruitment, and, although hypothesis testing was inhibited by small numbers of patients with a qSOFA score of 2/3, no clear signal was observed in biomarker levels between those with a score of 0/1 as compared to those with 2/3 (Supplementary Figs 1–3).

**Figure 3:**
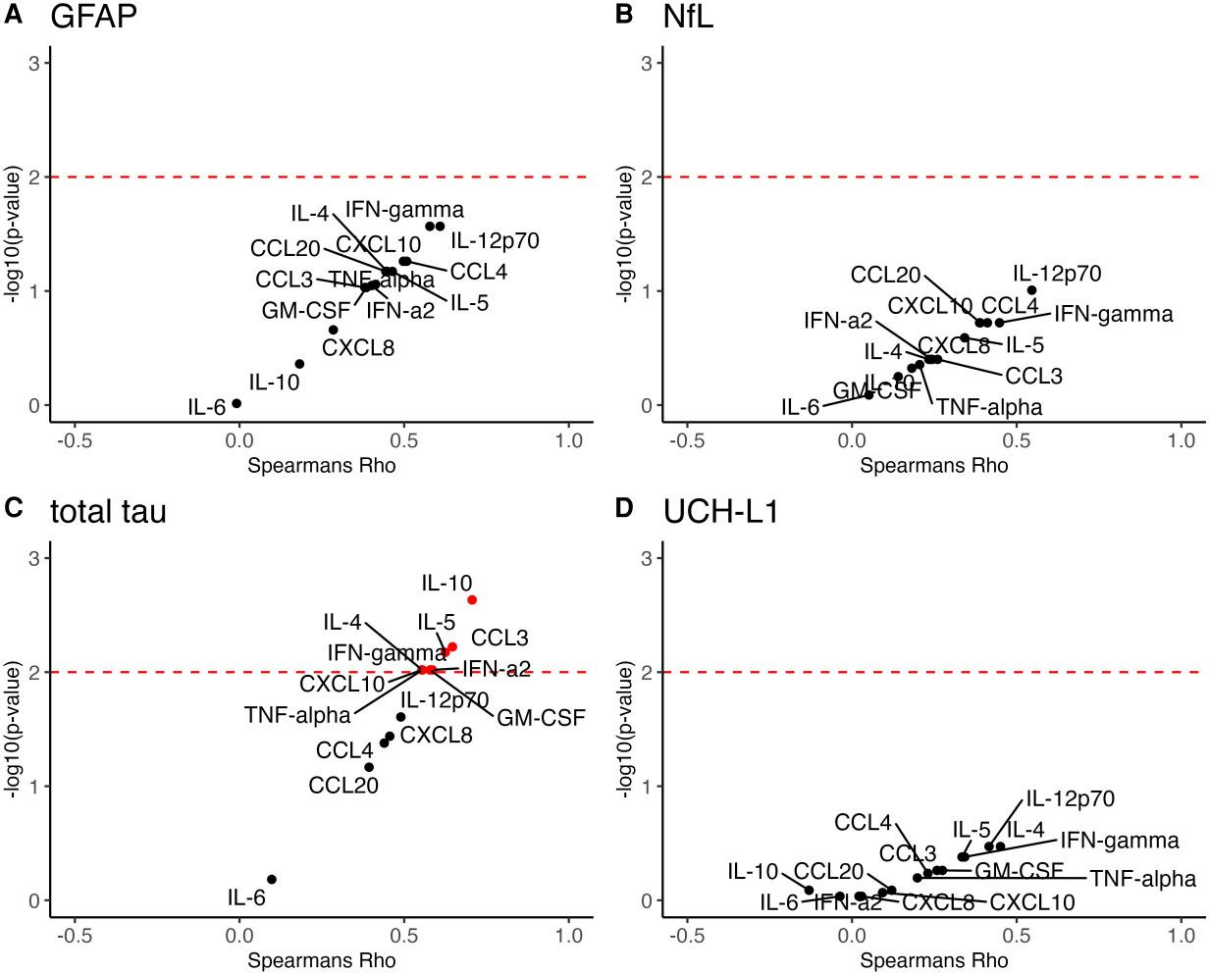
**A volcano plot of the spearman’s rank correlation coefficient and adjusted *P* values (false discovery method) of the correlation between biomarker and cytokine levels in patients with suspected sepsis (n = 23).** Volcano plots showing Spearman correlations between plasma biomarkers and cytokine levels in participants with suspected sepsis (n = 23). Panels show: (**A**) log GFAP (pg/ml), (**B**) log NfL (pg/ml), (**C**) log total tau (pg/ml) and (**D**) log UCH-L1 (pg/ml). In each panel, the x-axis represents the Spearman correlation coefficient (ρ) between the biomarker and cytokines, and the y-axis represents the FDR-adjusted *P*-value transformed as -log10, such that higher values indicate stronger statistical significance. Each point represents a single biomarker-cytokine pair. Black points indicate non-significant correlations, whilst red points indicate correlations with FDR adjusted *P* < 0.01. The red dashed line indicates the nominal significance threshold at -log10(0.01) = 2. S100B = S100 calcium-binding protein B.

In general, positive correlations were observed between biomarker levels and cytokines. Following adjustment for multiple comparisons, there were significant positive correlations observed between total tau and several individual cytokines (IL-4, IL-5, IL-10, CCL3, CXCL10, IFN-a2, GM-CSF, IFN-gamma, TNF-alpha) (Fig. 3). There were no significant associations between GFAP, NfL or UCH-L1 and any individual cytokines (Fig. 3). When the scaled cytokine values were entered into the principal component analysis algorithm, the first component accounted for 40% of the variance (Supplementary Table 1, Supplementary Fig. 4), with the subsequent loading plot of the first two dimensions shown in the Supplementary material (Supplementary Fig. 5). A significant association was observed between the summary inflammatory cytokine score and both GFAP and total tau (GFAP: ρ=0.53, *P* = 0.009; total tau ρ=0.65, *P* < 0.001), with no significant association seen with respect to NfL (ρ=0.34, *P* = 0.11) or UCH-L1 (ρ=0.33, *P* = 0.12).

In the sensitivity analysis to assess for differences by treatment allocation in the ABC-Sepsis trial, there were no significant differences in biomarker levels between the allocation groups (HAS versus crystalloid all *P* > 0.05). There was no significant elevation in total tau levels in patients with suspected sepsis with a pulmonary disease (n = 11) listed on discharge diagnosis as compared to those without (W = 82.5, *P* = 0.147). There was no significant correlation between plasma total tau levels and FiO_2_ on recruitment (ρ=−0.28, *P* = 0.200).

### Phosphorylated tau biomarker

**Figure 4:**
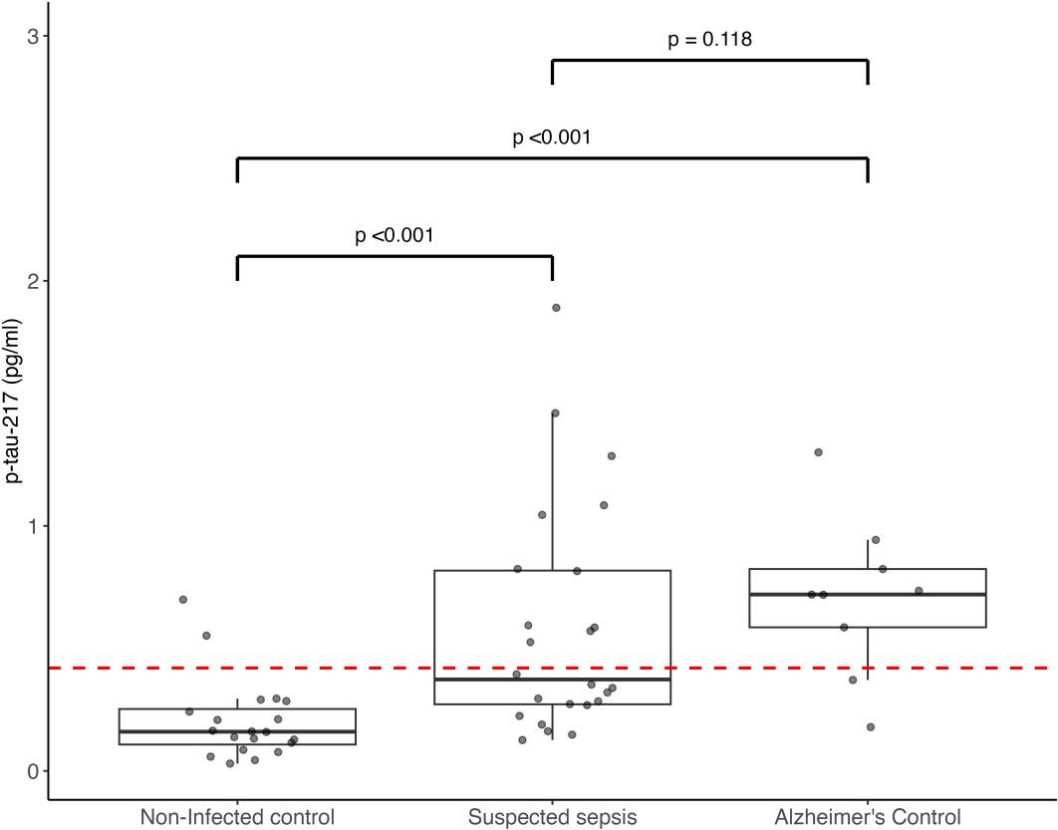
**Boxplots of the p-tau-217 levels in patients with suspected sepsis (n = 24) taken on recruitment, age- and sex- matched non-infected controls (n = 20) and Alzheimer’s disease patients (n = 9).** Boxplots showing the univariate distribution of plasma p-tau-217 (pg/mL) levels in participants with suspected sepsis (n = 24), age- and sex-matched non-infected controls (n = 20), and patients with Alzheimer’s disease (n = 9). Each box represents the IQR with the median shown as a horizontal line. Individual participant data points, each representing a single participant’s biomarker measurement, are overlaid as black dots. The red dashed line indicates the reported binary threshold for Aβ-positive positron emission tomography scan in Alzheimer’s disease (0.42 pg/ml).^16^ A Kruskal–Wallis test showed significant differences across groups (χ^2^ = 20.886, df = 2, *P* < 0.001). Post-hoc pairwise comparisons using Dunn’s test with Benjamini–Hochberg adjustment showed: Alzheimer’s disease versus non-infected controls, Z = 3.957, *P* < 0.001; suspected sepsis versus non-infected controls, Z = −3.713, *P* < 0.001; Alzheimer’s disease versus suspected sepsis, Z = 1.187, *P* = 0.118.

There was no correlation between total tau levels and levels of p-tau-217 in patients with suspected sepsis (ρ=0.22, *P* = 0.226). A Kruskal–Wallis rank sum test revealed a significant difference in p-tau-217 levels among patients with suspected sepsis, Alzheimer’s disease patients and non-infected controls (χ^2^ = 20.89, df = 2, *P* < 0.001). Pairwise comparisons showed a significantly higher level of p-tau-217 in patients with suspected sepsis and non-infected controls (*P* < 0.001) and between Alzheimer’s disease patients and non-infected controls (*P* < 0.001) (Fig. 4). There was no significant difference in levels of p-tau-217 in patients with suspected sepsis as compared to Alzheimer’s disease controls (*P* = 0.118). The results were unchanged when excluding suspected sepsis patients with a history of CNS disease (history of stroke, n = 3) (Kruskal–Wallis test: χ^2^ = 19.75, df = 2, *P* < 0.001, suspected sepsis versus non-infected controls *P* < 0.001, suspected sepsis versus Alzheimer’s disease controls *P* = 0.077).

There was no significant difference between patients with suspected sepsis and non-infected controls in p-tau-217:tau ratio (W = 135, *P* = 0.801) (Supplementary Fig. 6). There were no significant associations following adjustment for multiple comparisons between p-tau-217 and the levels of individual cytokines (Supplementary Fig. 7), with no significant association between p-tau-217 levels and the summary inflammatory cytokine score (ρ = 0.28, *P* = 0.18).

**Figure 5:**
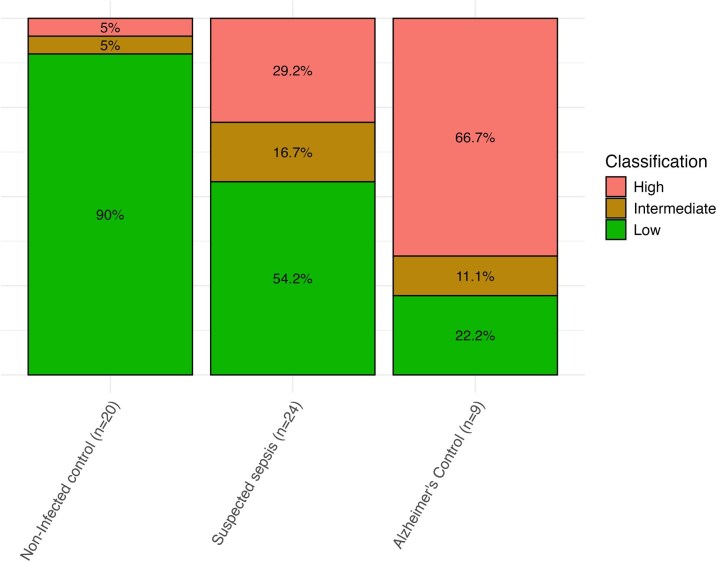
**Stacked bar graphs of the numbers of patients in each group with p-tau-217 levels in different reference ranges, demonstrating that nearly half of the patients with suspected sepsis had elevated plasma p-tau-217.** Distribution of plasma p-tau-217 concentrations (pg/ml) categorized as low (<0.4 pg/ml), intermediate (0.4–0.63 pg/ml) and high (>0.63 pg/ml) across participant groups: non-infected controls (n = 20), patients with suspected sepsis (n = 24) and Alzheimer’s disease controls (n = 9).^16^ The stacked bars show the proportion of participants in each category, with percentages annotated within each segment.

Of all non-infected controls with p-tau-217 results available, 90% had levels of p-tau-217 classified as low in reference to diagnostic ranges for Alzheimer’s disease, whilst 67% of Alzheimer’s disease controls had levels classed as high (>0.63 pg/ml). Of the patients with suspected sepsis, all without a prior diagnosis of neurodegenerative disease, 29% had a p-tau-217 level classified as high with a further 17% classified as intermediate (0.4–0.63 pg/ml) (Fig. 5).

Amongst patients with suspected sepsis, there was a significant correlation between age and p-tau-217 levels (ρ = 0.53, *P* = 0.008). The mean age of patients with suspected sepsis classified into the low p-tau-217 group was younger (mean ± SD 12) than the intermediate (77 ± 16 years) and high p-tau-217 group (78 ± 9 years); however, this difference was not statistically significant (ANOVA, (F(2, 21) = 2.61, *P* = 0.098) (Supplementary Table 2). Patients in the high p-tau-217 group had a higher mean lactate and a longer total length of stay in hospital (Supplementary Table 2).

## Discussion

In this analysis investigating blood-based biomarkers of neuronal and glial integrity and neurodegenerative biomarkers in a population with suspected sepsis, we identified elevated plasma total tau levels in suspected sepsis, associated with cytokine changes. There was similar, but less marked, elevation in plasma NfL levels suggesting a degree of neuronal damage in patients with suspected sepsis. However, there was no elevation in astrocyte (GFAP) or ubiquitin–proteasome (UCH-L1) markers and only minimal associations between these biomarkers and cytokine levels. There was no evidence of association between any brain injury biomarkers and markers of illness severity including admission lactate, NEWS2 score or qSOFA score. Additionally, we observed elevated plasma p-tau-217 levels during the acute phase of infective illness, with approximately 30% of patients with suspected sepsis and without a diagnosis of dementia or neurodegenerative disease exhibiting raised p-tau-217 levels.

It is important to consider the composition of the patient cohort in this analysis. Although all patients had an elevated NEWS2 score and clinical suspicion of infection, only one patient died within 90 days of recruitment and only two required admissions to critical care. This indicates that the cohort was less severely ill compared to populations in prior studies of brain injury biomarkers in sepsis, which often focus on critically ill patients with higher mortality rates.^7,13,24-28^ While most patients in this analysis had a discharge diagnosis consistent with an infectious illness, only five had positive blood cultures, characterizing this as a mixed infectious cohort. The comparison between our clinical population and healthy controls offers a unique perspective within the context of existing literature, thus demonstrating that even in comparatively mild sepsis/infection, there is an elevation of brain injury biomarkers. This elevation may indicate an element of subclinical brain injury following infection, even in the absence of critical illness or clinically observed neurological symptoms.

However, the disproportionate elevation of total tau as compared to neuronal, glial or ubiquitin–proteasome markers observed in patients with suspected sepsis is noteworthy. In cases of sepsis-associated encephalopathy, previous studies found higher serum total tau levels in patients with neurological symptoms compared to those without, consistent with the brain as a key source of tau rather than peripheral production.^26^ However, in reference to this analysis, if tau release was solely secondary to blood–brain barrier disruption or neuronal damage, one would expect a clearer relationship between total tau and other brain injury biomarkers; we did not observe a consistent pattern of elevation across these biomarkers, pointing away from a single cranial source of tau. This is further supported by the relationships between total tau and inflammatory mediators, with many cytokines showing significant positive associations with total tau but not with other biomarkers. These findings indicate an association between systemic inflammation and neuronal injury or tau dysregulation. However, whether this is due to tau elevation following cytokine increase, or a shared underlying mechanism, remains unclear. Overall, our findings suggest the presence of an extracranial source for tau, with an association between tau levels and infection or its physiological effects, including the host immune response.

Although predominantly thought of as a brain-specific marker, there are multiple possible sources of extracranial tau expression, with the most evidence relating to pulmonary expression. Animal models have demonstrated the release of cytotoxic tau proteins from lung epithelium in response to bacterial infection, leading to detectable levels of tau in the blood.^22,23^ In humans, the presence of cytotoxic tau has been found in the circulation of critically ill patients on extracorporeal membrane oxygenation with pneumonia, compared to those without.^22^ Additionally, elevations in total tau have been observed in bronchoalveolar lavage fluid from pneumonia patients, with a correlation between lavage tau levels and end-organ damage.^11^ Although 73% of the patients in our analysis were receiving oxygen therapy at recruitment, we observed no significant elevation in total tau between patients with a discharge diagnosis of pulmonary disease and those without, nor any significant correlation between admission FiO_2_ levels and total tau. While this was a post-hoc analysis, the lack of association with pulmonary pathology argues against the lung as the sole origin of the hypothesized extracranial tau release.

Elevations in total tau levels have been observed in athletes 1 h after strenuous exercise without concussion or sub-concussive impacts.^29-32^ While our study population did not undergo athletic exercise, they experienced many of the same physiological processes as a result of the systemic inflammatory response to illness, including sympathetic activation, and elevation in core body temperature, heart rate and respiratory rate. Additionally, we observed significant positive correlation between cytokines and total tau levels in our analysis, which is noteworthy given that acute bouts of strenuous exercise are known to trigger the release of pro-inflammatory cytokines.^33^ This raises the question of whether the total tau elevations observed in patients with suspected sepsis are a physiological response to physical stress or a pathological response to an infective trigger. Nevertheless, the findings of our study and the multiple potential extracranial sources of tau detailed in the wider literature indicate that care needs to be taken when interpreting total tau solely as a CNS-specific biomarker.

We observed elevations in p-tau-217 in patients with suspected sepsis, with levels significantly higher than age- and sex-matched controls. Approximately one-third of the cohort had p-tau-217 levels greater than the threshold that returns 95% specificity for amyloid β-positivity^16^ with no significant difference observed between the suspected sepsis cohort and Alzheimer’s controls in relation to p-tau-217 levels. To the best of our knowledge, this is the first assessment of p-tau-217 in the context of acute infectious illness or any extracranial acute illness. Other phosphorylated tau epitopes, such as p-tau-181, have been shown to be elevated in those with cognitive symptoms following SARS-COV-2 infection.^18,19,34^ Furthermore, p-tau-181 elevations have been observed in athletes after physical exertion, even without concussion or sub-concussive head impacts, raising again the possibility that elevation of phosphorylated tau is seen in response to exertion and from an extracranial source.^30^

Our findings of increased p-tau-217 in individuals with acute infective illness compared to age- and sex-matched non-infected controls are important despite the exploratory nature of the analysis, particularly in the context of current p-tau-217 research and its future clinical applications. Firstly, this is the first study to evaluate p-tau-217 in the context of infectious disease, and the potential for acute elevations of p-tau following infection maybe relevant to the infectious hypothesis of neurodegenerative disease. Secondly, the elevation of p-tau-217 in acute illness, whether due to the infection itself or the physiological stresses of illness, suggests that the presence of acute illness may be an important confounder to consider when interpreting p-tau-217 results. This has important implications for the timing of sampling and the interpretation of p-tau-217 results in clinical settings. Such considerations are particularly relevant when considering potential opportunistic sampling of p-tau in patients admitted to hospital for an acute illness, who may also have a background of gradual cognitive decline, where clinicians are interested in determining whether an underlying dementia process is present.

Neuropathological changes in keeping with Alzheimer’s disease are seen in cognitively normal older adults,^35^ which may influence p-tau-217 levels. In our suspected sepsis cohort, patients in the intermediate and high p-tau-217 groups were older on average than those in the low group, with positive correlation seen between age and p-tau-217. It may be that the greater p-tau-217 levels seen in the older adults with suspected sepsis reflects the higher baseline burden of p-tau-217 in the brain, and the disproportionate increase represents the leakage of this across the blood–brain barrier. Conversely, extracranial sources remain a possibility with greater physiological impacts of infection on individuals with greater degrees of frailty.

Previous studies have shown that elevations in p-tau-181 following SARS-CoV-2 infection are linked to brain structural changes and subsequent decreased cognitive scores.^19^ However, as our work represents a secondary analysis of the ABC Sepsis study and was limited to the outcomes originally collected in that cohort,^21^ we do not have long-term outcome or cognitive data for the patients, so the clinical impact of elevated p-tau-217 levels remains unclear. Prospective studies would be needed to explore the associations between acute infection, phosphorylated tau epitopes and long-term patient outcomes to better understand the complex causal pathways between these factors.

There are several limitations to this analysis. Notably, as an exploratory study of a single cohort, the conclusions should be interpreted with caution, and replication in a larger separate cohort would be needed to validate and expand upon these findings. We encountered assay failure in 10 samples when assessing the neurology 4-plex assay, including seven from the control group, which further reduced the sample size, increasing the risk of a Type II error. This limitation, especially when considering the small initial sample sizes, may have further reduced the statistical power to detect weaker associations and increased uncertainty around the strength and reproducibility of biomarker/cytokine correlations. Therefore, all findings should be viewed as preliminary and hypothesis generating, with replication required in larger, adequately powered prospective studies. Furthermore, owing to these sample size limitations, potential confounding by other co-existing conditions, including renal failure, cardiovascular history or obesity, could not be adequately addressed in our analysis and may influence biomarker results.^36,37^ Biomarkers were taken on recruitment, which occurred within 12 h of ED attendance; however, the duration of illness prior to ED presentation could not be standardized. This may dilute the signal, especially for the cytokine panel, where different cytokines are elevated at varying time points following infection. However, whilst these issues limit the interpretation of the biological link between infection and brain injury, they strengthen our finding that interpretation of, e.g. Alzheimer’s, biomarkers is context specific and based on our data, not reliable during acute illness. Although blood sampling beyond the acute phase of illness was not available from the MIS-ABC Sepsis study, future studies assessing longitudinal biomarker profiles in patients with infectious disease represent an important opportunity to evaluate changes following resolution of the acute infection and over the longer term. Participants with suspected sepsis were defined as those with clinically suspected or proven infection, a NEWS or NEWS2 score ≥ 5. Owing to the absence of consistent microbiological or definitive clinical confirmation, misclassification is possible, with some participants potentially having lower-severity infection and two participants having discharge diagnoses of non-infectious conditions that mimicked a clinical sepsis presentation.

Further, owing to the method of recruitment for the study, there was no baseline pre-morbid cognitive testing, p-tau-217 sampling or amyloid β positron emission tomography. Therefore, the estimations of undiagnosed Alzheimer’s disease pathology in the cohort are limited to clinical diagnosis prior to study recruitment. This means we cannot assess changes in biomarker levels from participants’ own baseline values. Additionally, given the prolonged preclinical phase of Alzheimer’s disease that can last for years before clinical diagnosis, some patients with suspected sepsis may be in this preclinical stage (indeed, this may be a risk factor for infection).^38^ This could influence the results, as the observed biomarker changes may reflect underlying preclinical Alzheimer’s disease rather than being solely induced by infection. Despite matching for age and sex, we cannot exclude that the BioResource controls are not systematically different to those who present to the ED following infection. These differences may introduce sampling bias, including a higher chance undiagnosed Alzheimer’s disease. However, the lack of GFAP elevation in the suspected sepsis cohort would point away from this owing to its known utility in differentiating between controls and pre-clinical Alzheimer’s disease with elevations in GFAP observed in peripheral blood samples years prior to Alzheimer’s diagnosis.^39^

## Conclusion

In this analysis of patients with suspected sepsis, we have identified elevated levels of total tau and NfL compared to age- and sex-matched controls, along with significant correlations between these tau levels and cytokine levels. Additionally, we observed elevated levels of p-tau-217 in the patient cohort, with levels comparable to those seen in Alzheimer's disease patients. These findings suggest a potentially extracranial source of tau expression in the context of infection or physiological stress. Given the potential for acute illness to influence p-tau-217 levels, our results raise important considerations regarding the interpretation of p-tau-217 as a diagnostic marker for Alzheimer's disease in patients with active infection. Further research is necessary to clarify the mechanisms underlying these observations and to determine the clinical relevance of p-tau-217 in the context of acute illness.

## Supplementary Material

fcag063_Supplementary_Data

## Data Availability

Data are available from the corresponding author upon reasonable request. R code is publicly available at: https://github.com/danw1310/Brain_communications_code.
