# Supplementary material for: Brain-injury and Alzheimer’s disease biomarkers are elevated in patients with suspected infection and physiological derangement: importance for context-specific interpretation of Alzheimer’s biomarkers
Source: Brain Commun. 2026 Feb 28;8(2):fcag063. doi: 10.1093/braincomms/fcag063 (PMC12978425; doi:10.1093/braincomms/fcag063)
Supplement: fcag063_Supplementary_Data [file fcag063_supplementary_data.pdf]

Supplementary Data: Brain-injury and Alzheimer’s disease biomarkers are elevated in patients with suspected infection and physiological derangement: importance for context-specific interpretation of Alzheimer’s biomarkers.

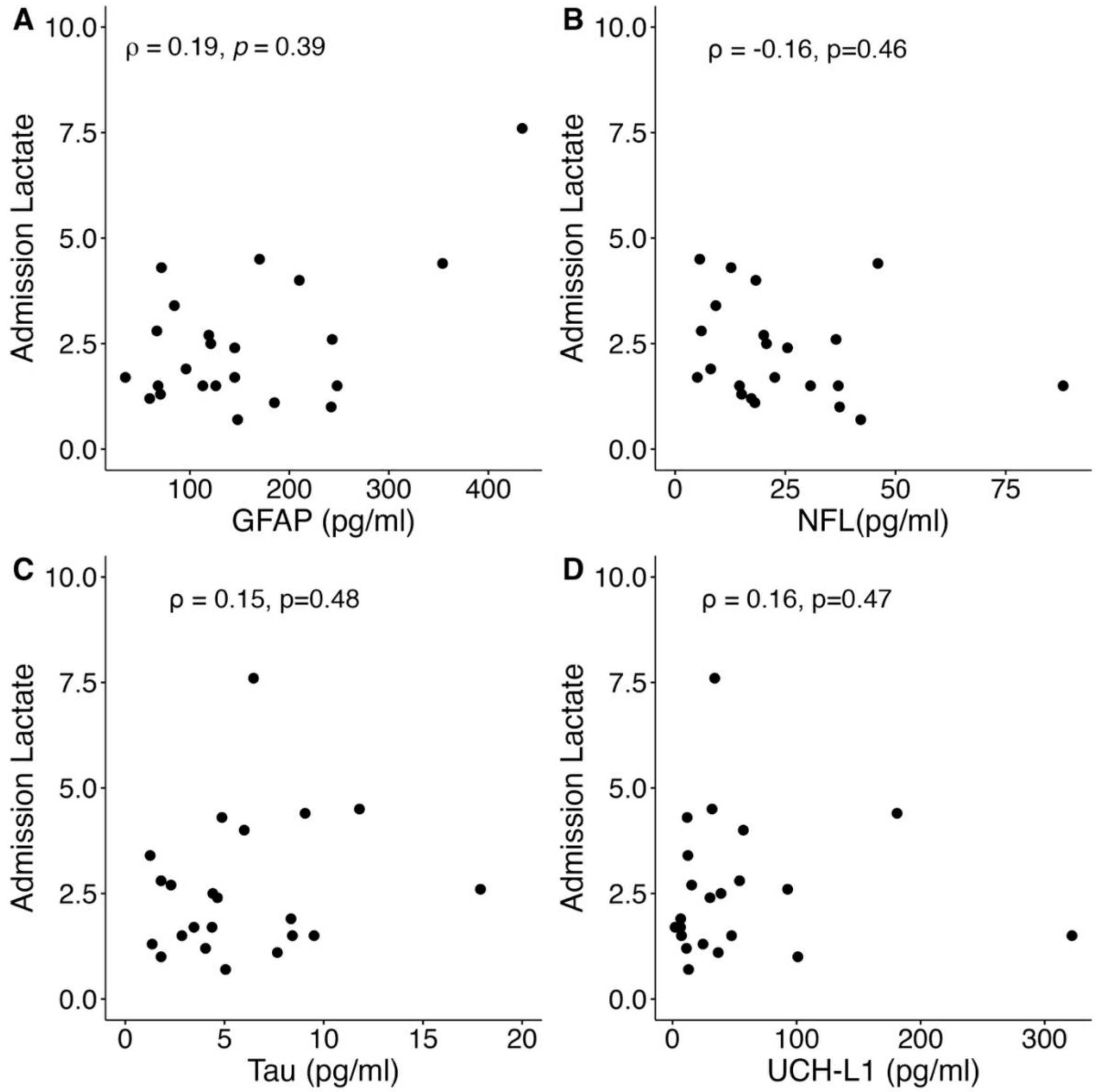

Supplementary Figure 1. Scatter plots showing the correlation between plasma biomarker concentrations and admission lactate levels in patients with suspected sepsis (n = 23). Panels show: A) GFAP (pg/ml), B) NfL (pg/ml), C) total tau (pg/ml), and D) UCH-L1 (pg/ml). Each data point represents a single participant. Spearman's rank correlation coefficient (ρ) was used to assess the association between biomarkers and lactate levels. Two extreme outliers (NfL = 219 pg/ml; total tau = 267 pg/ml) were omitted from the plots for graphical clarity; all statistics were calculated including these values. Test results were as follows (Spearman's rho, p value): GFAP: $\rho = 0.19$, $p = 0.39$; NfL: $\rho = -0.16$, $p = 0.46$; total tau: $\rho = 0.15$, $p = 0.48$; UCH-L1: $\rho = 0.16$, $p = 0.47$.

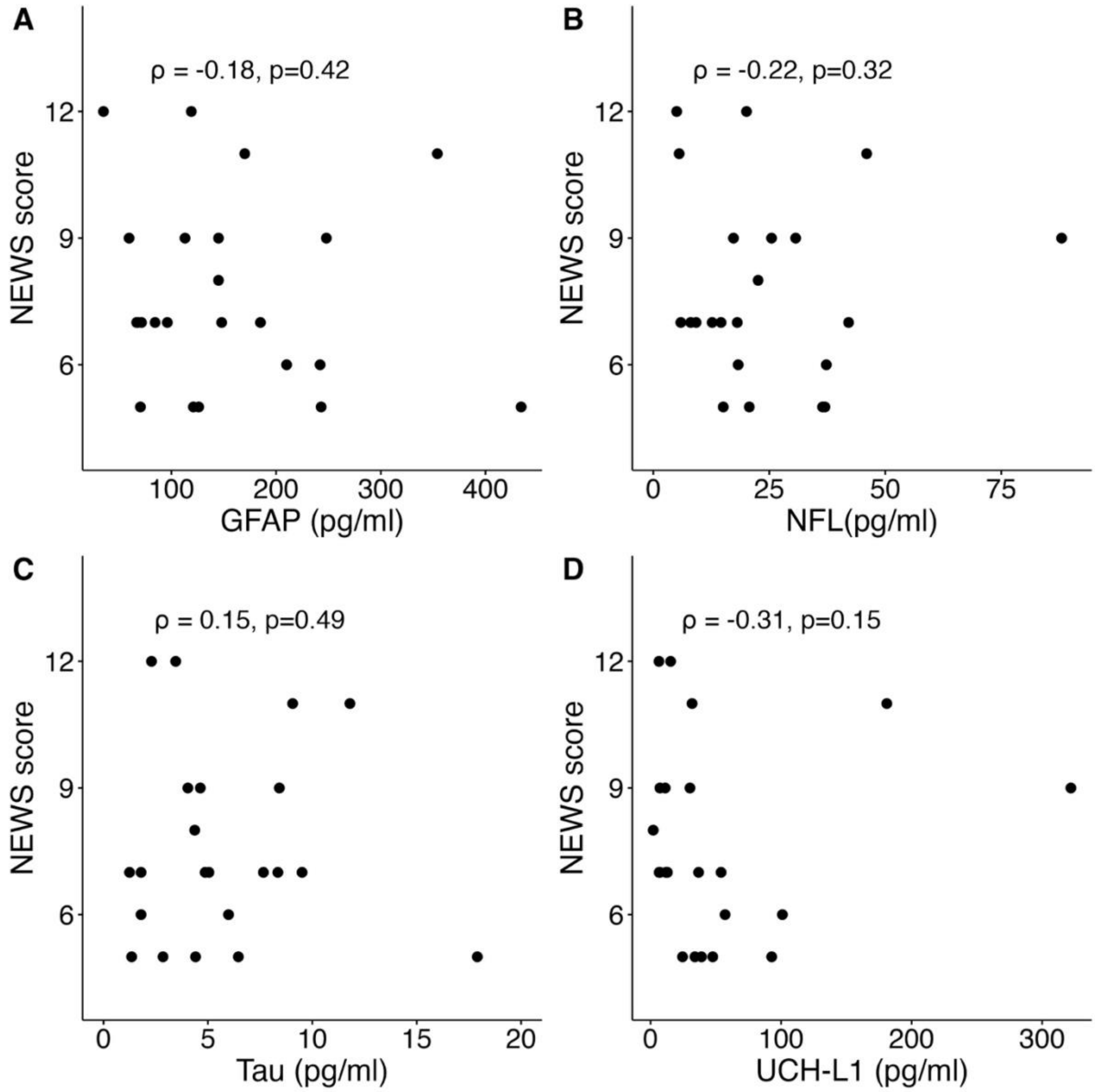

Supplementary Figure 2. Scatter plots showing the correlation between plasma biomarker concentrations and admission National Early Warning Score (NEWS 2) in patients with suspected sepsis (n = 23). Panels show: A) GFAP (pg/ml), B) NfL (pg/ml), C) total tau (pg/ml), and D) UCH-L1 (pg/ml). Each data point represents a single participant. Spearman's rank correlation coefficient (ρ) was used to assess the association between biomarkers and NEWS score. Two extreme outliers (NfL = 219 pg/ml; total tau = 267 pg/ml) were omitted from the plots for graphical clarity; all statistics were calculated including these values. Test results were as follows (Spearman's rho, p value): GFAP: ρ = -0.18, p = 0.42; NfL: ρ = –0.22, p = 0.32; total tau: ρ = 0.15, p = 0.49; UCH-L1: ρ = -0.31, p = 0.15.

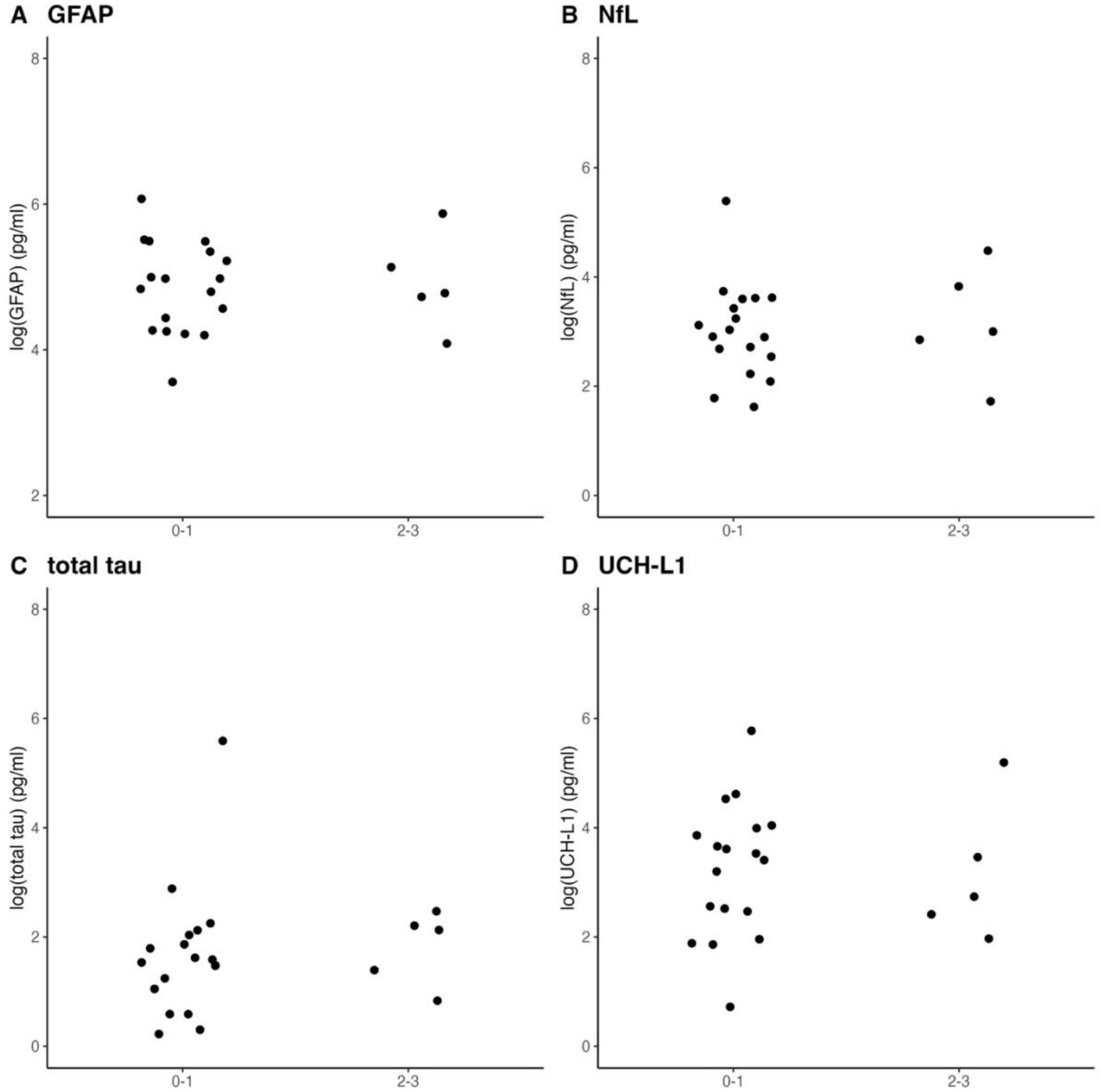

Supplementary Figure 3. Dot plots of biomarker levels and quick Sequential Organ Failure Assessment Score (qSOFA) score dichotomised as 0 or 1 (n = 18) vs 2 or 3 (n = 5) in patients with suspected sepsis. Panels show: A) GFAP (pg/ml), B) NfL (pg/ml), C) total tau (pg/ml), and D) UCH-L1 (pg/ml). Each data point represents a single participant. Log-transformed biomarker concentrations are shown on the y-axis.

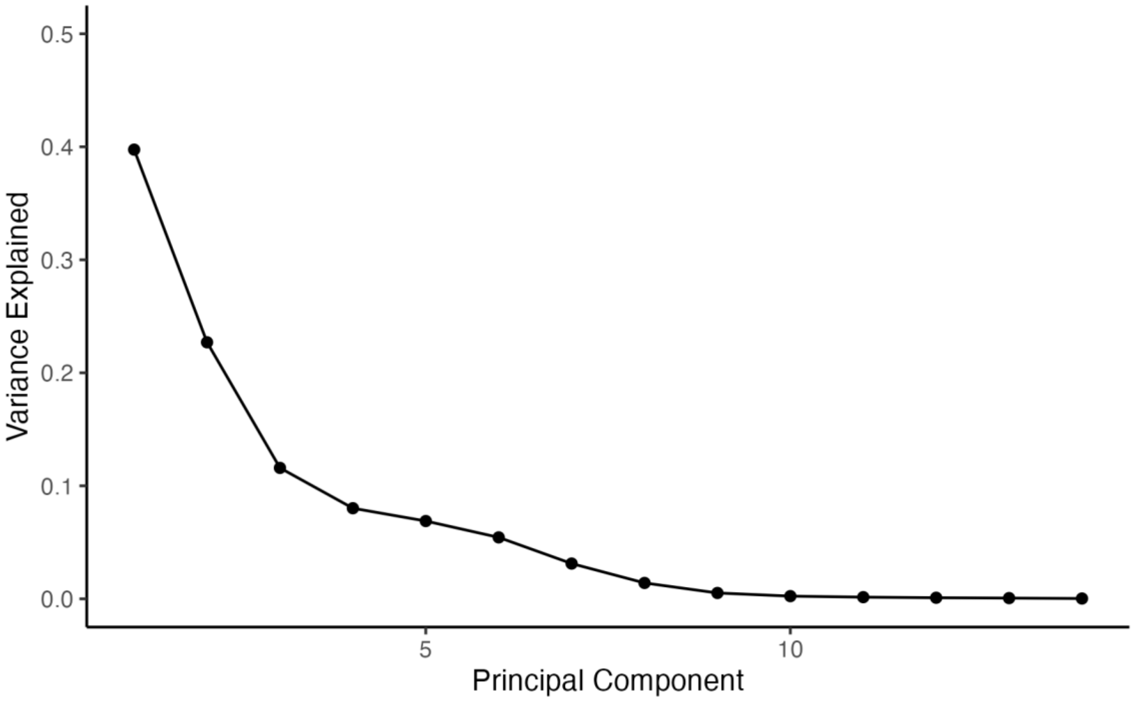

Supplementary Figure 4. Scree plot showing the proportion of variance explained by each principal component in the Principal Component Analysis (PCA) of inflammatory cytokines in patients with suspected sepsis (n=26). The scree plot shows the proportion of variance explained by each principal component derived from the PCA of inflammatory cytokine levels. All variables were scaled prior to PCA.

Supplementary Table 1. Principal Component 1 Loadings for Inflammatory Cytokine Levels

	**PC1 Loadings**
**TNF-alpha**	0.37230
**IL-6**	0.10312
**GM-CSF**	0.38798
**CXCL10**	0.14423
**IL-5**	-0.00533
**IFN-a2**	0.35592
**IL-4**	0.37820
**CCL3**	0.38762
**IL-10**	0.01575
**IL-12p70**	0.13917
**CCL20**	0.12553
**IFN-gamma**	0.35365
**CXCL8**	0.30350
**CCL4**	0.08296

Standardised factor loadings for each of the 14 inflammatory cytokines contributing to the first principal component (PC1) from all patients with suspected sepsis (n=26). PC1 represents the linear combination of cytokine variables that explains the greatest proportion of total variance in the inflammatory profile. Higher absolute loadings indicate a stronger contribution of the corresponding cytokine to PC1.

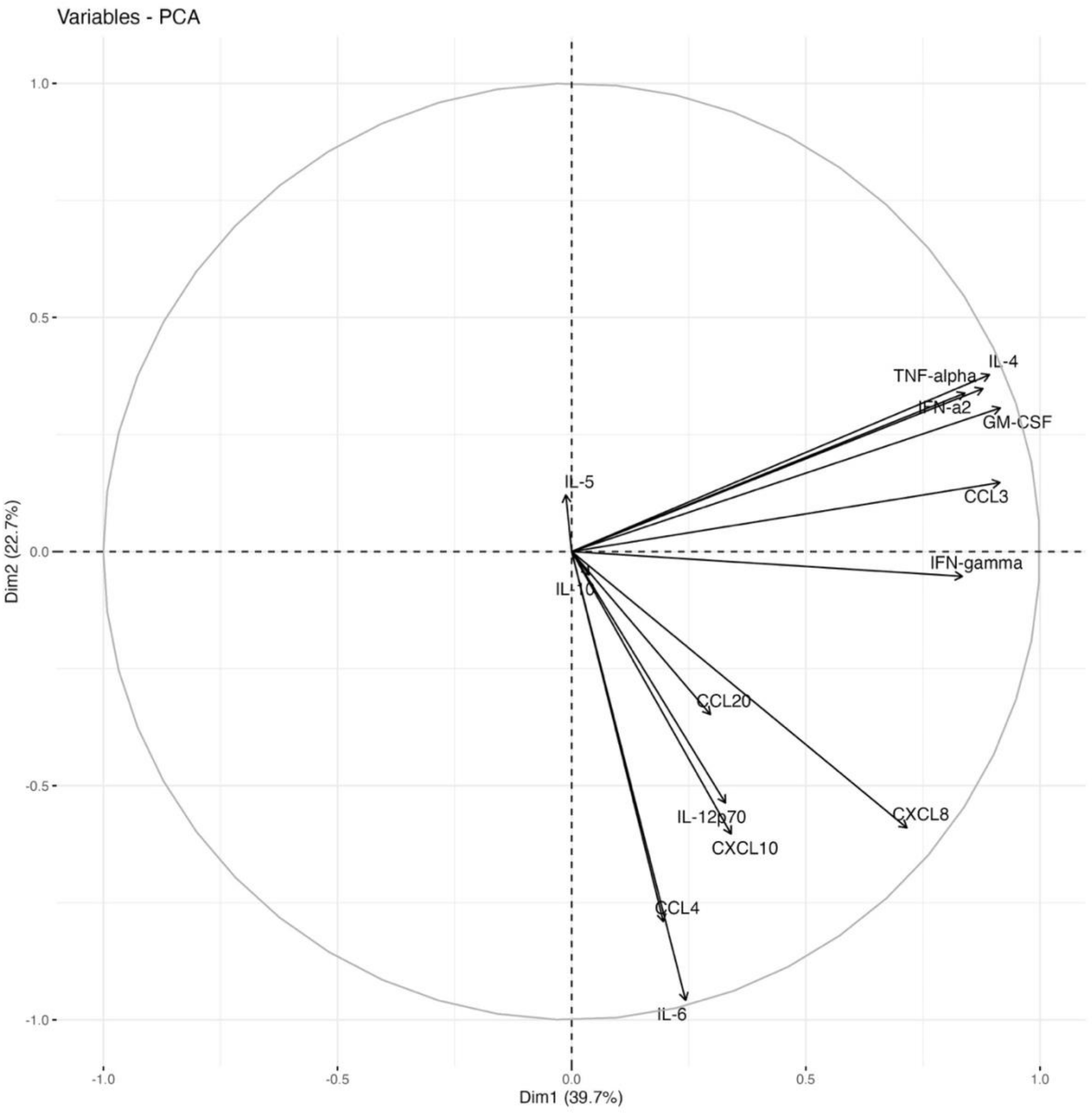

Supplementary Figure 5. Loadings plot of the principal component analysis of cytokine values. The plot shows the variable loadings of individual cytokines on the principal components derived from PCA (n=26). Cytokine values were scaled prior to analysis, and only complete cases were included. The direction and magnitude of each vector indicate the contribution and correlation of each cytokine to the principal components, with longer vectors representing stronger contributions.

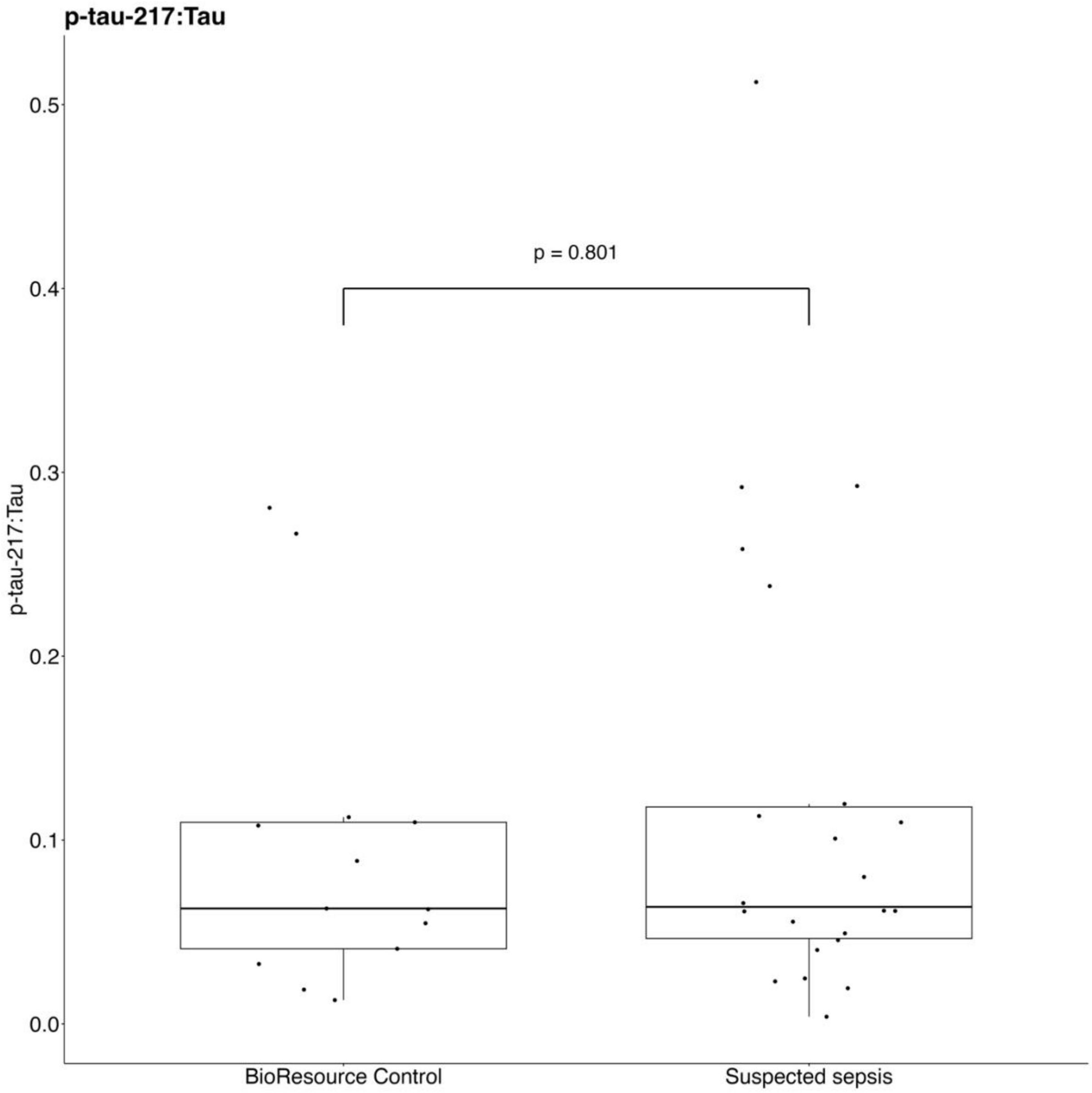

Supplementary Figure 6. Box plot of the p-tau-217 (pg/ml):tau (pg/ml) ratio in patients with suspected sepsis (n=22) and non-infected age and sex matched controls (n=13). Each box represents the interquartile range (IQR) with the median indicated by a horizontal line, and individual participant data points overlaid as black circles, each representing a single participant. Horizontal bars indicate the statistical comparison between groups (suspected sepsis vs non-infected age- and sex-matched controls), with the p-value annotated above. Group comparisons were performed using the Wilcoxon rank-sum test (W = 135, p = 0.801).

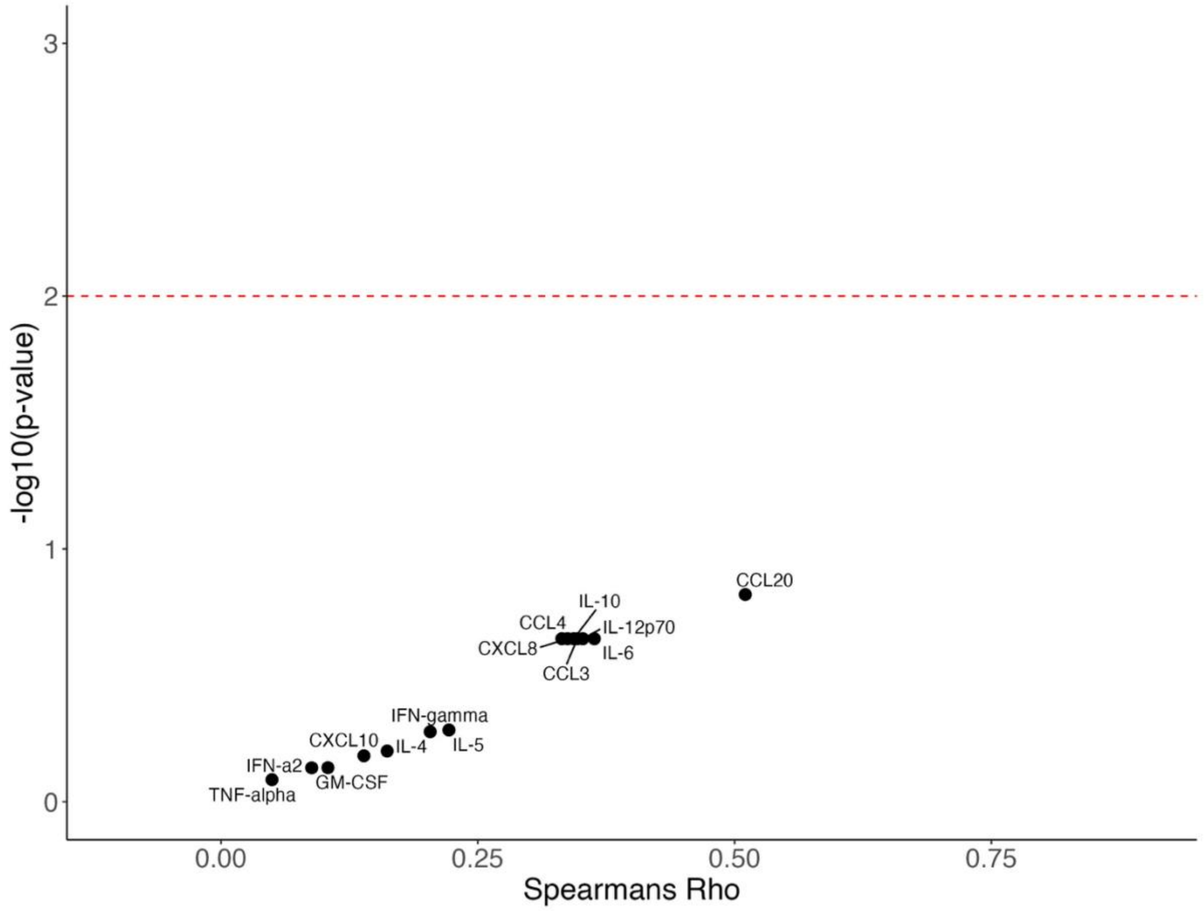

Supplementary Figure 7. A volcano plot of the Spearman's rank correlation coefficient and adjusted p values (false discovery method) of the correlation between p-tau-217 and cytokine levels in patients with suspected sepsis (n=24). The x-axis represents the Spearman correlation coefficient (ρ) between the biomarker and cytokines, and the y-axis represents the FDR-adjusted p-value transformed as -log10, such that higher values indicate stronger statistical significance. Each point represents a single biomarker-cytokine pair. The red dashed line indicates the nominal significance threshold at -log10(0.01) = 2. p-tau-217 = phosphorylated tau 217.

Supplementary Table 2. Demographics of patients with suspected sepsis as separated by p-tau-217 category (n=24).

	High (n= 7)	Intermediate (n=4)	Low (n=13)	p
**Age (years)**	78 (9)	77 (16)	66 (12)	0.098
**Sex (Male)**	5 (71.4)	3 (75.0)	4 (30.8)	0.122
**NEWS score (median)**	7.00 [5.00, 10.00]	7.00 [6.75, 8.25]	7.00 [7.00, 9.00]	0.959
**Lactate at presentation (mmol/L)**	3.03 (2.26)	1.48 (0.88)	2.35 (1.30)	0.314
**Glasgow Coma Score (%)**				0.483
**13**	1 (14.3 %)	0 (0.0 %)	0 (0.0 %)	
**14**	1 (14.3 %)	1 (25.0 %)	1 (7.7 %)	
**15**	5 (71.4 %)	3 (75.0 %)	12 (92.3 %)	
**Total length of stay (days)**	23.86 (26.60)	6.00 (2.83)	9.15 (9.35)	0.116
**90-day survival (%)**	7 (100.0)	4 (100.0)	12 (92.3)	0.643

Means (SD) of continuous variables unless otherwise stated, Count (%) of categorical variables. NEWS = National Early Warning Score. P values determined by ANOVA test for continuous variables and chi-squared test for categorical.
